# Physical Literacy, Physical Activity, and Health: A Citation Content Analysis and Narrative Review

**DOI:** 10.1186/s40798-025-00827-8

**Published:** 2025-04-25

**Authors:** Kathryn Fortnum, Meyene Duque Weber, Dean Dudley, Eloisa Tudella, Matthew Kwan, Veronique Richard, John Cairney

**Affiliations:** 1 The University of Queensland, Health and Wellbeing Centre for Research Innovation, School of Human Movement and Nutrition Sciences, Brisbane, QLD Australia; 2 Department of Physiotherapy, The Federal University of São Carlos, São Carlos, Brazil; 3 Macquarie School of Education, Macquarie University, Macquarie Park, NSW Australia; 4 Department of Family Medicine, Infant and Child Health Lab, McMaster University, Hamilton, Canada

## Abstract

Physical literacy has received increased research attention over the last decade focusing on the unification of the definition, measurement, and application, including in school and health-based contexts. In 2019, Cairney et al. released a model positioning physical literacy holistically as a primary determinant of health and disease, mediated by physical activity (PA), the physiological and psychological adaptations associated with PA, and the individual and social/environmental/contextual factors or conditions that impact PA-related behaviour, which had a significant impact on physical literacy-related literature. To assess the impact of the model on the extant literature, and better understand the relationship between physical literacy, PA and health as proposed by Cairney et al., we conducted a citation content analysis and narrative review. 956 citations were identified citing the model proposed by Cairney et al. Of these, 16 used the model to construct a theoretical framework and were included in the extended analysis. Thirteen studies were observational, and participants were all children or young people with a total age range 4–20 years. Results demonstrate that physical literacy is related to health-related fitness variables including aerobic fitness, body composition, flexibility, and muscular strength and power; total PA and MVPA; and health literacy, and wellbeing, supporting the model proposed by Cairney et al. However, gaps remain in understanding critical components of the model (e.g., the proposed mediation pathways), and in clarifying the nature of the relationships in a variety of populations (e.g., clinical populations) and across the lifespan. A pragmatic approach to addressing these gaps is recommended.

## Key Points

- Physical literacy has been positioned as a determinant of health, with prominent physical literacy researchers including John Cairney proposing models that link physical literacy to health; however, the impact of such models in the literature, and the relationships proposed in the models are not well understood.
- Based on literature using the model proposed by John Cairney and colleagues in 2019, we demonstrate that physical literacy is associated with physical activity, as well as health and wellbeing outcomes.
- Our understanding of the relationships between physical literacy, physical activity and health is beginning to emerge; to enhance our understanding, research focusing on the potential influencing factors proposed in the model, and that includes clinical populations across a broad range of ages is recommended.

## Introduction

Physical literacy is a multidimensional construct that includes affective, social, physical, and cognitive domains [14]. The application, operationalisation, and conceptualisation of physical literacy in the literature, however, is varied and contested. More of the recent holistic conceptualisations will include some combination of constructs including motor competence (e.g., movement skills, sport specific skills, fitness, and strength), psychosocial elements (e.g., positive affect, physical activity-related motivation, confidence and self-competence, and social competence), and cognitive elements (e.g., understanding of how and why to be active, and sports-specific knowledge) [1–8]. Physical literacy is an inclusive concept that can be applied to people of all ages from children and adolescents [9] to older adults [10] and is considered a lifelong journey that is inherently part of an individual’s identity, and unique life experiences [1, 8].

Physical literacy has received increased research attention over the last decade. One major area of physical literacy-based research pertains to the unification of the definition of physical literacy. This research is driven by the persistent diversity in the conceptualisation, application, and operationalisation of the foundations and determinants underpinning physical literacy. Examples of such studies include those by Jurbala [11] and Edwards et al. [12]. A second major area of research focuses on the measurement of physical literacy, which is important for increasing understanding of the concept and facilitating its translation from a theoretical or philosophical to operationalisable concept [4]. Two current, yet paradigmatically opposed, approaches to the measurement of physical literacy are the *idealist* or ‘academic’ and *pragmatic* or ‘practical’ perspectives [4, 13]. Idealists view physical literacy as a holistic concept that can only be measured as a whole, and any attempt to de-aggregate the construct into its constituent parts is resisted [11]. Conversely, those adopting a pragmatic perspective recognise that physical literacy measurement must align with evidence-based measurement practices, and that an evidence-based approach to measurement is essential to change current practices [14, 15].

The increased research attention on the measurement of physical literacy has enabled further applications of the construct within the literature. Research on physical literacy in specific or traditionally-marginalised population groups (e.g., people living with disabilities [14, 16] or with mental health disorders [17], or new migrants [18]) and contexts (e.g., physical education, and athlete development [12, 19]) is gaining traction. The application of physical literacy also extends to research focusing on interventions theoretically underpinned by physical literacy, or that which focuses on physical literacy as an intervention outcome. A recent systematic review and meta-analysis by Carl et al. [20] examining the outcomes of interventional research underpinned theoretically by physical literacy, or examining physical literacy as an outcome, demonstrated significant positive effects on either the individual physical literacy components, total physical literacy, and/or PA levels. The examination of physical literacy within applied observational and experimental research is a critical step towards the practical application and adoption of physical literacy into education, sport, and public health practices [3, 21].

**Fig. 1:**
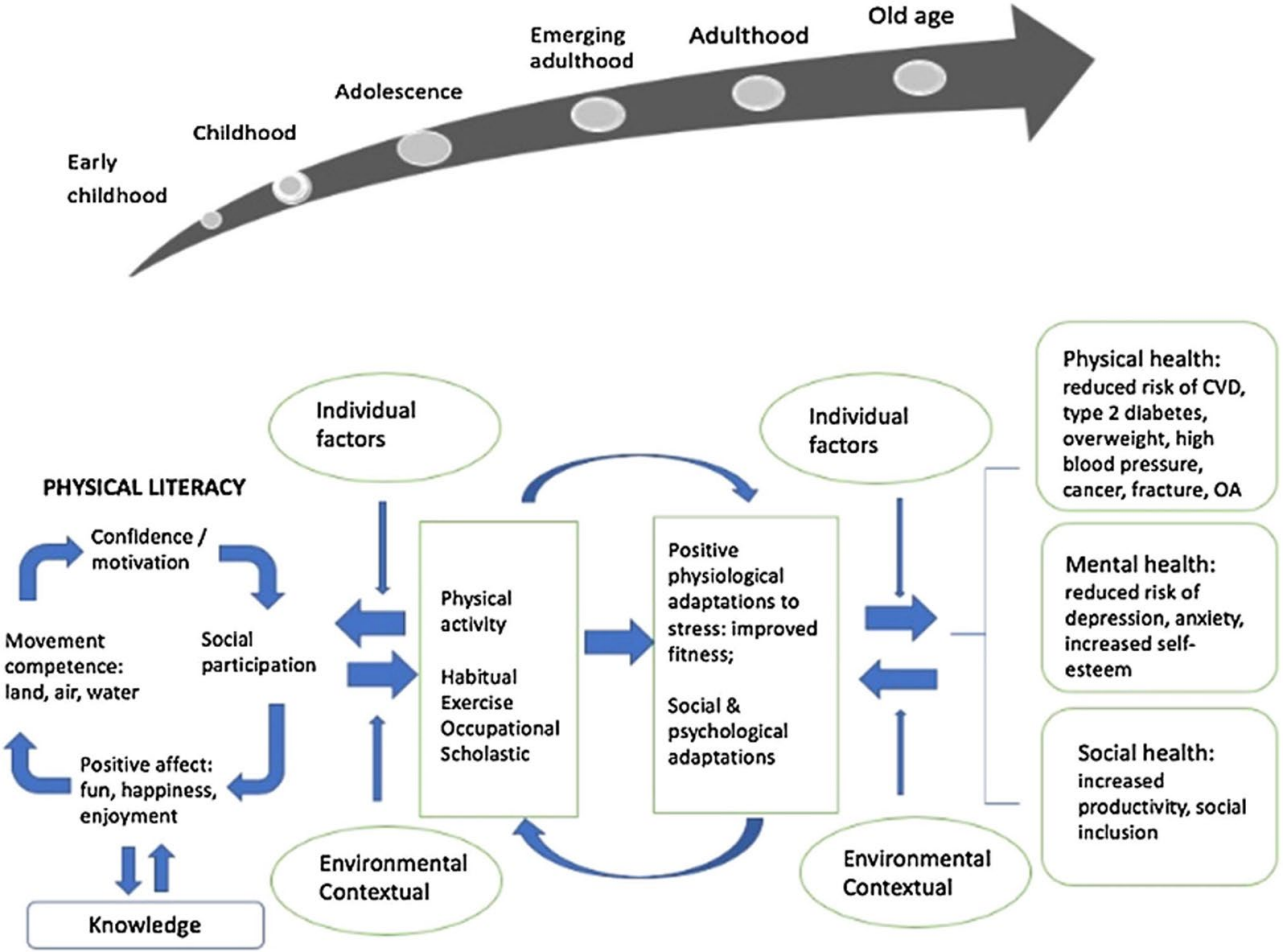
Conceptual model linking physical literacy, physical activity and health proposed by Cairney et al. [22]. CVD = Cardiovascular disease, OA = Osteoarthritis

Combining evidence highlighting the links between physical literacy and PA with the well-founded understanding of the positive impacts of PA on health-related outcomes; a logical next step for physical literacy-related research is to consider the links between physical literacy, PA, and health-related outcomes. While several papers have drawn an explicit connection between physical literacy and health [2, 11, 22], one model has gained significant attention in the literature since its publication. Cairney et al. [22] position physical literacy as a primary determinant of health and disease, which is both mediated by PA, the physiological and psychological adaptations associated with PA, and further influenced by the individual and social/environmental/contextual factors or conditions that impact PA-related behaviour (see Fig. 1). Physical literacy itself is depicted as a reciprocal, inter-related feedback loop linking confidence/motivation, social participation, positive affect to movement competence, with knowledge arising as an outcome of the interaction among those elements.

Cairney et al. [22] utilised the example of children diagnosed with developmental coordination disorder (DCD)—which has a hallmark feature of impaired motor coordination—as a case study to demonstrate the physical literacy and health link. In children with DCD, the combined effect of poor motor competence, negative affect and motivation towards PA is accompanied by poorer fitness and physical and mental health-related concerns. Cairney et al. [22] also noted the influence of social factors commonly experienced by children with DCD (e.g., social isolation, teasing, bullying) on PA participation, and physical and mental health.

The model proposed by Cairney et al. [22] has made a significant impression on physical literacy-related literature. According to a bibliographic analysis conducted by Mendoza-Muñoz et al. [23], the work of Cairney et al. [22] received the second highest number of citations compared to all other physical literacy-focused articles, including those published by prominent physical literacy researchers. Though highly cited, the impact of the model within physical literacy-related literature has not yet been fully explored. For example, as the most comprehensive/holistic model of physical literacy, it is important to investigate how physical literacy-focused research and theory has been influenced by the model and to what extend empirical tests of the model have been undertaken. Addressing these queries would help with; (a) establishing trends in physical literacy research and (b) identifying relationships—within the model—that remain under explored.

In this paper, we first conducted a literature review to identify publications citing Cairney et al. [22]. Secondly, to assess how the model is being used in the extant literature, we conducted a citation content analysis (CCA) of identified publications [24, 25]. This research method was chosen since it is widely used to assess the impact and the substance of scientific production [24, 25]. Finally, to obtain clarification about the potential relationships between physical literacy and health, and the mediating/moderating factors influencing this relationship, we conducted a narrative analysis to synthesise empirical studies referencing the model.

## Main Text

### Methods

#### Stage 1: Literature Review

To examine the impact of the model, a three-stage approach was adopted. In Stage 1, citations that referenced Cairney et al. [22] were retrieved. The search was conducted using the ‘cited by’ function in PubMed, Web of Science, Scopus, and Google Scholar. Citations from inception up to July 2023—the date of conducting the search—were included. Grey literature was also searched in ProQuest, Altmetric, and Google databases using the article title and author names. There were no restrictions placed on publication types. All citations were imported to Covidence™ software to check for duplicates. Subsequently, the same citations were imported into EndNote for secondary verification of duplicate citations. The full text of all remaining citations was identified for screening. If the full text could not be identified, corresponding authors were contacted. Authors were provided with one week to respond, after which a follow-up email was sent. If no response was received after an additional week, the publication was excluded (n = 7). The inclusion criteria for Stage 1 were as follows; (1) publications written in English, Spanish or Portuguese; and (2) publications citing Cairney et al. [22]. No additional inclusion or exclusion criteria were applied within Stage 1. Dimensions AI was used to graphically capture publication country and field of research codes (FoR, Australian and New Zealand Standard Research Classification [26]) for articles meeting the inclusion criteria. Publications meeting the above-mentioned inclusion criteria progressed to Stage 2.

#### Stage 2: Citation Content Analysis

Zhang et al. [25] describes eight syntactic codes (Sy; A–H) which provide a representation of how data are presented and organised within the cited-citing relationship. Codes relate to either the cited publication (A–F) or citing (G–H). *A. Type of documents cited* refers to the type of publication being cited. In this case Cairney et al. [22] is the only publication being considered, so ‘Journal article’ was the only applicable value*. B. Type of authorship* (Sy) pertains to the number of authors on the publication being cited and has the options of either single or multiple authors. *C. Relation to the citing work* (Sy) allows for the study of the latent connection between cited and citing works, with three options available, specifically reciprocal, parallel or hierarchical. Reciprocal refers to self-citation; parallel refers to citing peers, co-authors, or collaborators; and hierarchical refers to citing prestigious authors with high social capital. Cairney J. has been recognised in multiple papers as a significant contributor to physical literacy-related research [27, 28]; therefore, was considered to have high social capital. *D. Location of mentioning* refers to where in the citing publication Cairney et al. [22] was mentioned or referenced (e.g., abstract, introduction, methodology, results/discussion, conclusion). *E. Frequency of mentioning* refers to the number of times Cairney et al. [22] was referenced in the citing publication. *F. Style of mentioning* (i.e., not specifically mentioning, specifically mentioning but interpreting, or direct quotation) was used to indicate the potential importance of the Cairney et al. [22] within the referencing publication, as a citation can be considered more, or less, relevant depending on the extent and depth to which it is discussed. *G. Type of citing documents* refers to the type of document citing Cairney et al. [22] (e.g., journal article, conference paper, book/book chapter). *H. Type of Authorship* refers to the number of authors of the citing document.

Zhang et al. [25] also describe four semantic (Se) codes (I–L) used to describe what data are being presented. I–J relate to Cairney et al. [22] and K–L to the citing publication*. I. Function of citation* describes the application of Cairney et al. [22] within the citing article with three options; (a) provide background information (i.e., the citation usually appears in the introduction or literature review sections and provides general contextual information); (b) construct a theoretical framework (i.e., the citation usually appears in the methods section and is associated with the hypothesis or theory underpinning the article's methodology); and (c) describe challenges and limits (i.e., the citation usually appears in the discussion or conclusion sections and is used to support or negate research findings). *J. Disposition of citation* elicits whether the citing publication referred to Cairney et al. [22] from a positive (i.e., acknowledgement), neutral (i.e., objective) or negative (i.e., questioning and challenging) standpoint. *K. Type of research domain* categorises the citing publication into one or more of social sciences, humanities, natural sciences, or applied sciences and engineering. The final code *L. Type of research focus* describes the focus of the citing article as either theoretical research, empirical research, experimental research or other.

In Stage 2 publications were initially categorised according to *I. Function of citation* of Cairney et al. [22, 25]. Categorisation was performed by one author (MDW), with a secondary review conducted by a second author (KF) for 20% of the publications plus any publications that could not be clearly categorised (agreement = 100%). To be included in Stage 3 of the analysis, publications must have used Cairney et al. [22] to construct a theoretical framework. The included publications were categorised into the remaining syntactic and semantic categories pertaining to the cited-citing interaction, as detailed by Zhang et al. [25] (detailed in Sect. "Stage 1: Literature Review" and summarised in Table 1). For categorisation in accordance with the codebook, NVivo software was utilised (Version 12.6.1 for Windows). The included articles were imported from EndNote into NVivo, and the software's tools assisted in the syntactic and semantic analyses presented in Table 1. As per the codebook, analysis was conducted either using ‘single-sentence level’ (i.e., only the sentence citing Cairney et al. [22]) or ‘sentence-cluster level’ (i.e., the 1–2 sentences surrounding the Cairney et al. [22] citation) [25].

**Table 1:** Codebook for citation content analysis [25] including the citing articles that used Cairney et al. [22] to construct a theoretical framework (n = 16)

Orientation	Categories	Values	n
*Syntactic (Sy)*			
Cited	Type of document cited	Journal article	16
		Conference paper	0
		Book/book chapter	0
		Report/news	0
		Link/personal blog	0
		Others	0
	Type of authorship	Single-authored	0
		Multiple-authored	16
	Relation to the citing work	Reciprocal (self-citation)	3
		Parallel (cite co-author)	0
		Hierarchical (cite-author with high social capital)	13
	Location of mentioning (n = number of citations within articles)	Abstract	0
		Introduction	27
		Literature Review	0
		Methodology	5
		Results/discussion	7
		Conclusion	1
		Others (specify)	0
	Frequency of mentioning	Once	3
		2 to 4 times	11
		5 times or more	2
	Style of mentioning	Not specifically mentioning	2
		Specifically mentioning but interpreting	14
		Direct quotation	0
Citing	Type of citing documents	Journal article	16
		Conference paper	0
		Book/book chapter	0
		Report/news	0
		Link/personal blog	0
		Other	0
	Type of authorship	Single-authored	0
		Multiple-authored	16
*Semantic (Se)*			
Cited	Function of citation (n = number of citations within articles)	Provide background information	3
		Construct theoretical framework	36
		Provide previous empirical/experimental evidence	0
		Describe challenges and limits	0
	Disposition of citation (n = number of citations within articles)	Positive	0
		Negative	0
		Mixed	0
		Neutral	39
Citing	Type of research domain	Social sciences	16
		Humanities	2
		Natural sciences	0
		Applied sciences and engineering	16
	Type of research focus	Theoretical research	0
		Empirical research	16
		Experimental research	0
		Other (specify)	0

‘n’ refers to the number of articles unless otherwise specified. Reproduced from Zhang et al. [25], with permission

#### Stage 3: Narrative Review

To allow for the examination of empirical evidence pertaining to the model proposed by Cairney et al. [22] additional data were extracted for the included articles, specifically author details, publication country, participant details (e.g., age, sex, health conditions), outcome assessment tools, and main results. These data were synthesised using narrative methods [29].

### Results

#### Literature Review and Citation Content Analysis

**Fig. 2:**
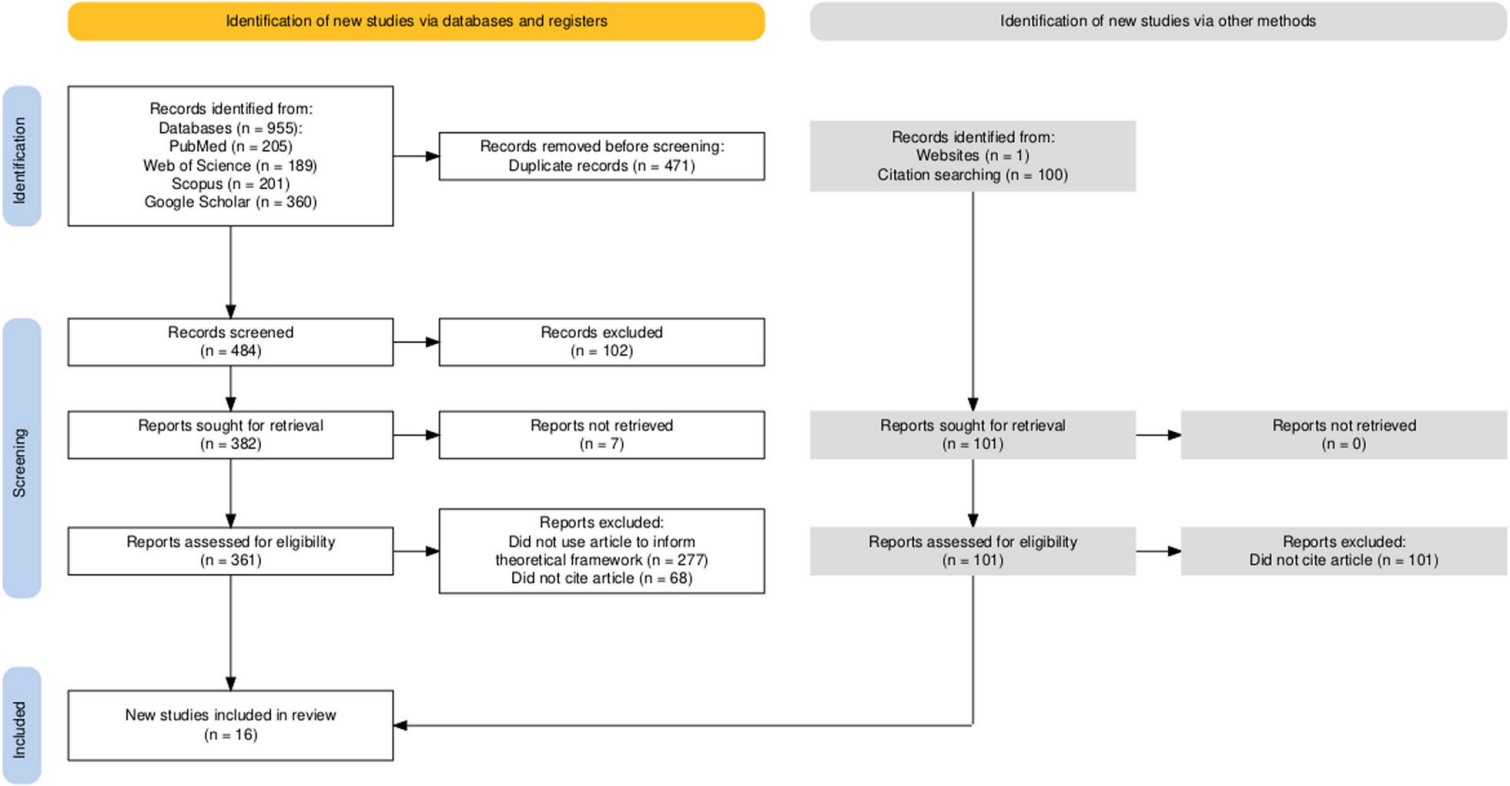
PRISMA Flow diagram [30]. Citation searching in ‘identification of new studies via other methods’ refers to ProQuest

The initial search returned 1056 publications (PRISMA flow diagram available in Fig. 2)—955 from citation functions in databases, one from a website, and 100 from ProQuest (dissertations and theses). Following removal of duplicates, 484 remained, of which 102 were excluded as they were not written in English, Spanish or Portuguese. A final 361 articles, 100 dissertations and theses, and one website were assessed for eligibility. Of these, 68 articles, all 100 dissertations and theses and the one identified website did not cite Cairney et al. [22] so were removed from any further analysis. A final 293 studies were eligible for progression into Stage 2.

**Fig. 3:**
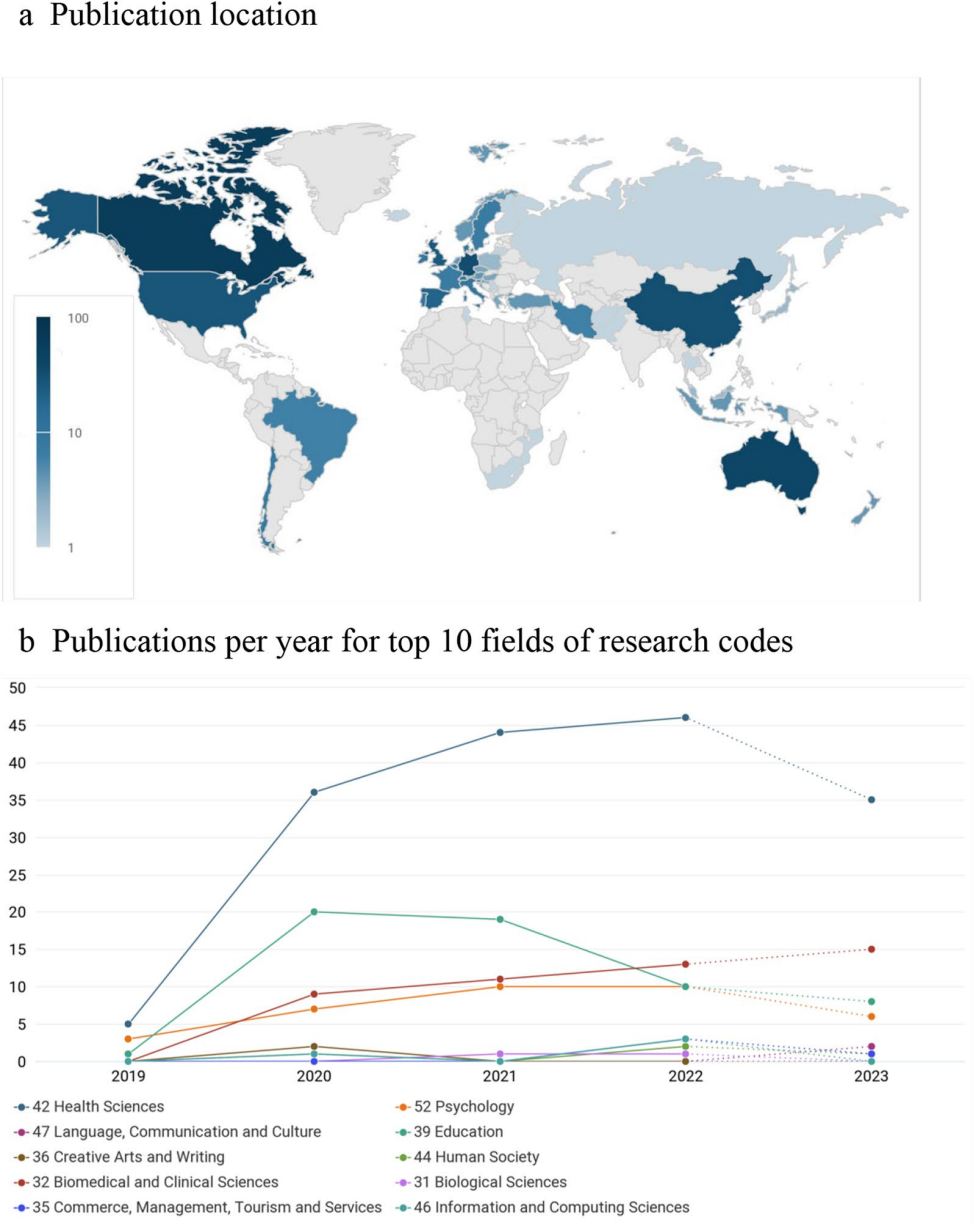
Dimensions AI analytics for 247 of the total 293 eligible citations*.* (Note: 2023 includes publications up to July 2023 only). **a** Publication location, **b** Publications per year for top 10 fields of research codes

Only 247 of the 293 publications were available in Dimensions AI. Figure 3 provides graphical representation of the countries the studies citing Cairney et al. [22] originated from as well as the publication dates for the top FoR codes, as generated by Dimensions AI. The top five citing countries were Canada (n = 62), Australia (n = 40), Germany (n = 33), China (n = 32) and the United States (n = 27).

Sixteen of the 293 eligible studies used Cairney et al. [22] to construct a theoretical framework and were included in the extended analysis. Characterisation of the included studies following the CCA codebook defined by Zhang et al. [25] is presented in Table 1. Cairney et al. [22] was mentioned or referenced most in the introduction section (n = 27 citations), with most articles (n = 11) referencing Cairney et al. [22] 2–4 times. Citing articles typically interpreted meaning from Cairney et al. [22] to provide study-specific background or context. All citing articles adopted a neutral disposition towards Cairney et al. [22]. Additional study detail and key results are available in Table 2.

**Table 2:** Study details and key results from all studies included in the extended analysis

Study details	Study design	Population	Outcomes (and measurement tools) in alignment with PL model	Key results
*Experimental studies*				
Arbour-Nicitopoulos et al. [14]	Quasi-experimental	n = 67 children in grades 6–12 (mean age 12.2 yrs, 31.3% girls) with or without a disability (ambulant or use a wheelchair for ambulation, able to move independently)	PL: Ignite Challenge, BREQ-3, PA self-efficacy scale, Peer MCYSQ (end of program only) PA: Accelerometry	Significant findings following 16 wk group PL program focusing on exposure to fun games and skills: Substantial improvement in motor competence Small improvement in motivation (relative autonomy index, integrated motivation and amotivation) Increase in moderate PA (medium effect) Decrease in MVPA and vigorous PA (medium effect)
Kwan et al. [31]	Quasi-experimental	n = 65 first year University students (n = 46 women, mean age 17.85yrs) Control n = 39, intervention n = 26	PL: PLAYfun, PA-related self-efficacy (Bandura), BREQ-3 PA: IPAQ-SF Health: Standing long jump test (muscle power), grip strength, 20 m shuttle run (aerobic fitness),	Significant outcomes following 12 week intervention focusing on novel movement skills: MVPA: No change in intervention group, ↓ in control group Aerobic fitness: ↑ in intervention group, ↓ in control group Predicted CRF: ↑ in intervention group, no change in control group PL did not have a mediation effect on any of the above results
Nezondet et al. [32]	Quasi-experimental	n = 13 adolescents (mean age 11.7 yrs, 31% girls)	PL: CAPL-2 PA: CAPL-2, Accelerometry Health: BMI, Body composition (BIA), 20 m shuttle walk/run (aerobic fitness)	Significant findings following 9-month educational program designed to increase PL: Strong, positive correlation between PL and VO2peak; PL and MVPA; and MVPA and VO2peak
*Observational Studies*				
Brandelli et al. [38]	Cross-sectional	n = 25 (48% female) children aged 4–10 yrs with juvenile idiopathic arthritis (JIA)	PL: PLAYparent, PLAYself, TGMD-3 PA: Accelerometry Contextual factors: Parent perceptions of PA-related risk *Health*^*a*^*: BMI, Childhood Health Assessment Questionnaire*	Significant effect of JIA joint involvement on FMS; Higher ball skills and total FMS scores correlated with increased MVPA Ratings of PL (parent and child) not significantly related to MVPA
Britton et al. [35]	Cross-sectional	n = 1073 children aged 9–14 yrs (57% female) involved in the Moving Well-being Well National PL study	PL: TGMD-3, BOTMP-2, PASES, BREQ-3 Health: Plank hold (strength), KIDSCREEN-27, PACER (aerobic fitness)	Factors contributing most to accuracy of predicting wellbeing (based on machine learning): Females: Object-control skills, identified motivation and confidence (balance and locomotor contribute to a lesser degree) Males: Confidence and balance (object control skills locomotor contribute to a lesser degree) Aged 9–10 yrs: All nine PL features contribute, object control skills, balance and intrinsic motivation contributed most Aged 11–12 yrs: VO2max (confidence, locomotor skills and introjected motivation contribute to a lesser extent)
Brown et al. [33]	Longitudinal cohort study	n = 2015 5th grade students (mean age 10.3 yrs; 49.4% girls)	PL: BOTMP-SF, SPPC, CSAPPA, additional 3 questions to explore perceptions of physical competence, PA motivation, and enjoyment of PA PA: Participation Questionnaire *Health*^*a*^*: BMI*	Profile analysis → 5 profiles: Inconsistent low PL defined by divergent very low enjoyment of PA Low PL Inconsistent low PL defined by divergent high enjoyment Moderate PL High PL Associations between profiles and PA: Profiles 4 & 5 associated with significantly greater PA levels than profiles 1–3 Profile 5 associated with significantly greater PA than profile 4
Caldwell et al. [40]	Longitudinal cohort study	n = 222 (n = 113 girls) ‘healthy’ children aged 8–13 yrs	PL: PLAYfun, PLAYself, PLAYparent PA: Accelerometry Health: BMI, %BF (BIA), blood pressure, Bruce protocol (aerobic fitness), PedsQL	PL composite score significantly related with each health indicator and MVPA Strongest association between PL and aerobic fitness; partly mediated by MVPA Relationship between PL and other health indicators not mediated by MVPA
Clark et al. [34]	Longitudinal cohort study	n = 2078 (49.2% female) students aged 10–12 yrs (grades 5–7)	PL: BOTMP-SF, SPPC, CSAPPA PA and SB: Participation Questionnaire	Profile analysis → 3 profiles: 1. Active Screeners (AS; high SB and high PA) 2. Low SB (low screen time, moderate to high PA) 3. Sedentary/inactive (SI; high SB, low PA) Associations between profiles and outcomes: Students with higher PL more likely to be in AS and Low SB groups than SI One unit increase in PL reduced odds of transitioning from AS to SI by ~ 50% for males and ~ 33% for females Students in SI had significantly higher BMIs (in grade 6) and significantly lower self-concept (in grade 7) compared to AS and Low SB
Gilic et al. [36]	Cross-sectional	n = 544 ‘healthy’ adolescents (n = 403 girls) aged 14–18 yrs	PL: PLAYself, CAPL-2-KU Health: BMI, standing long jump test (muscle power), sit-up test (muscle strength), sit and reach test (flexibility), beep test (aerobic fitness)	All: CAPL-2-KU with flexibility and muscle strength. PLAYself total with BMI, muscle power and strength & flexibility Girls: CAPL-2-KU with aerobic fitness. PLAYself total with BMI, muscle power and strength, flexibility & aerobic fitness Boys: CAPL-2-KU with muscle strength. PLAYself total with muscle power and strength, aerobic fitness
Kesic et al. [37]	Cross-sectional	n = 253 adolescents (n = 181 girls; aged 10–19 yrs) without acute inflammatory disease (e.g., COVID-19)	PL: PLAYself Health: HLS-EU-Q, BMI, body composition (BIA)	Boys: Small correlation of PL to fat mass Girls: Small correlations of PLAYself numeracy, PLAYself literacy and PLAYself PL to health literacy
Melby et al. [41]	Cross-sectional	n = 1518 adolescents (51.3% female) aged 13–15 yrs	PL: MyPL questionnaire PA: “How many hours do you normally use on sport/exercise per week (not counting time used on transportation)?” Health: Wellbeing composite score: Self-esteem (3 items), Cantril Ladder of life scale, Body Investment Scale, Loneliness (1 item)	All: Small correlations between PA and PL; PA and all wellbeing variables except self-esteem; and PL and all wellbeing variables Girls: Small correlations between PL and all wellbeing variables; and PA with wellbeing composite score and life satisfaction Boys: Small correlations between PL and all wellbeing variables; and PA and all wellbeing variables except self-esteem Higher β-coefficients for direct association between PL and wellbeing variables in girls compared to boys
Mendoza-Muñoz et al. [23]	Cross-sectional	n = 135 children (53.3% female) aged 8–12 yrs without pathologies that prevent participation in physical fitness tests or practice	PL: CAPL-2 PA: CAPL-2 Health: BMI, Fat mass (BIA)	Non-overweight children had higher scores in all PL domains (including daily behaviour i.e., PA) and total PL PL total score of overweight children predominantly ‘beginning’ and ‘progressing’ compared to ‘progressing’ to ‘excelling’ in non-overweight children Physcial competence predominantly ‘beginning’ in overweight children compared to ‘progressing’ to ‘excelling’ in non-overweight children Motivation and competence ‘beginning’ in overweight children compared to ‘progressing’ to ‘excelling’ in non-overweight children Small to moderate inverse correlation found between body composition and PL total score, and all PL domains except knowledge and understanding
Nezondet et al. [43]	Cross-sectional	n = 85 adolescents (mean age 12.1 yrs, n = 32 girls) able to complete all assessment items	PL: Perceived PL Instrument PA: YRBSS Health: BMI, Body composition (BIA), 20 m shuttle walk/run (aerobic fitness)	Small to moderate positive association between PL and % skeletal muscle mass; and MVPA and % fat mass (FM) Moderate negative association between PL and %FM; aerobic fitness with BMI; aerobic fitness and %FM Moderate positive association between PL and aerobic fitness; PL and MVPA; aerobic fitness and MVPA; and MVPA with % skeletal muscle mass Strong, positive correlation between aerobic fitness and %skeletal muscle mass MVPA mediated relationship between PL and % skeletal muscle mass and aerobic fitness
Öztürk et al. [39]	Cross-sectional	n = 568 University students (n = 405 women) aged 18–20 yrs without orthopaedic or neurological problems	PL: Perceived PL Instrument, PACES, Physical Activity Barriers PA: IPAQ-SF Contextual factors: Physical Activity Barriers	PA associated with PL total score, self-confidence, self-expression, and knowledge PL total score significantly different between highly active, moderately active, and inactive individuals (higher if more active) PL total score and self-expression sub-scale significantly higher for moderately active compared to inactive individuals PL self-confidence and knowledge sub-scales significantly higher in highly active compared to moderately active and inactive individuals PA impacted PACES total, positive and negative scores
Yan et al. [44]	Cross-sectional	n = 2996 College students (mean age 20.16 yrs, 72.3% female)	PL: Perceived PL Scale PA: IPAQ-SF Health: BMI	MVPA of participants who were normal weight and obese was significantly higher than those who were underweight PL of participants who were normal weight was significantly higher than participants who were obese Small positive correlation between PL and LPA; and PL and MVPA (partly mediated by SB and LPA) Small negative correlation between PL and SB; SB and LPA partly mediated by the relationship between PL and MVPA
Zhang et al. [45]	Cross-sectional	n = 798 University students (mean age 19.2 yrs, 51.1% female)	PL: Perceived PL Scale PA: IPAQ-SF Health: BMI, spirometry (vital capacity) 800 m/1000 m timed run (aerobic fitness), standing long jump (muscle power), sit and reach (flexibility)	Men and women: Confidence and physical competence correlated (small) with muscular strength and aerobic fitness; total PL correlated (small) with vital capacity and aerobic fitness Men: Motivation correlated (small) with aerobic fitness and vital capacity; MVPA negatively correlated (small) with vital capacity; MVPA positively correlated (small) with confidence and physical competence in men Women: MVPA positively correlated (small) with vital capacity in women; MVPA and muscular strength positively correlated (small) Participants categorised as ‘fit’ had higher PL than ‘unfit’- differences between fit and unfit groups for confidence and physical competence found for all fitness tests excluding vital capacity; differences between fit and unfit for motivation evident for aerobic fitness only; differences between fit and unfit for environmental interaction evident for vital capacity only

^a^ and italics = Assessed but not associated with physical literacy or physical activity

*PL* physical literacy; *PA* physical activity; *TGMD-3* test of gross motor development 3rd ed; *MVPA* moderate to vigorous PA; *BOTMP—SF* Bruininks–Oseretsky test of motor proficiency—short form; *SPPC* Harter’s self-perception profile for children; *CSAPPA* children’s self-perceptions of adequacy in and predilection for physical activity; *%BF* % body fat; *BIA* bioelectrical impedance analysis; *PedsQL* pediatric quality of life; *SB* sedentary behaviour; *CAPL-2-KU* Canadian assessment of physical literacy knowledge and understanding questions; *HLS-EU-Q* European health literacy survey questionnaire; *IPAQ—SF* international physical activity; questionnaire—short form; *CRF* cardiorespiratory fitness; *PACES* physical activity enjoyment scale; *LPA* light physical activity; *SB* sedentary behaviour; *MCYSQ* motivational climate youth sport questionnaire

#### Narrative Analysis—Experimental Studies

Participants included in the citing studies were all children or young people with a total age range 4 ~ 20 years. Three of the included 16 studies were experimental (all quasi-experimental). The experimental studies all utilised a physical literacy informed intervention and focused on outcomes assessing physical literacy and PA (n = 1) [14]; or physical literacy, PA, and health-related parameters (n = 2) [30, 31]. Following a 16-week group-based intervention, Arbour-Nicitopoulos et al. [14] found improvements in motor competence, motivation, and moderate PA (MPA) in children with a disability (ambulant or using a wheelchair for ambulation, but able to move independently) or without a disability. Nezondet et al. [31] reported a strong positive correlation between physical literacy, moderate-to-vigorous PA (MVPA) and aerobic fitness in adolescents without motor, mental, cognitive, or psychic disabilities or current injuries following a 9-month educational, therapeutic program. Kwan et al. [30] reported maintenance of MVPA levels and improvements in aerobic fitness in a group of university students who participated in 12-week novel-movement skill intervention compared to a decline in the control group who did not receive any intervention; however, the results suggested that they were not mediated by physical literacy.

#### Narrative Analysis—Observational Studies

The remaining 13 studies were observational (longitudinal n = 3, cross-sectional n = 10). The observational studies assessed the relationship between physical literacy and PA (n = 2) [32, 33]; physical literacy and health-related parameters (n = 3) [34–36]; physical literacy, PA, and contextual factors (n = 2) [37, 38]; or physical literacy, PA, and health-related parameters (n = 6) [39–44]. Only one study, Brandelli et al. [37] specifically included a clinical population group; juvenile idiopathic arthritis (JIA). They reported relationships between JIA and fundamental movement skill (FMS) development, whereby JIA joint involvement specifically, negatively impacted FMS development. Additionally, the authors reported a significant positive correlation between FMS and MVPA, and increased parent-ratings of ‘risk’ of a given activity to be associated with lower locomotor skills and lower parental-perceived physical literacy. Ratings of physical literacy (child and parent) and parental risk ratings were not associated with MVPA [37].

Synthesising results from the observational studies, it can be determined that; higher physical literacy (total and sub-scales e.g., self-confidence, self-expression) is associated with higher PA [32, 33, 38, 40, 43] (partly mediated by SB and LPA [43]) and lower SB [33, 43]. Physical activity enjoyment was also shown to be associated with PA levels [38]. A small to large relationship was evident between physical literacy and aerobic fitness, [35, 39, 42, 44] which was (partly) mediated by MVPA [40, 43]. Total physical literacy as well as the individual components (e.g., confidence, motivation, physical competence) were associated with flexibility, and strength, muscle power; associations that were gender specific [36]. Body composition was also related to physical literacy, whereby higher physical literacy was associated with improved body composition (i.e., lower BMI, lower % fat mass [35, 36, 41–43]]. Small correlations also existed between physical literacy and wellbeing variables [40]; and machine learning models indicated that movement skills and specific components of motivation and confidence predict wellbeing in children [34]. One study reported small correlations between physical literacy and health literacy [36].

### Discussion

Research focusing on physical literacy has increased in frequency in the last decade. One seminal paper that has received attention from many researchers is that published by Cairney et al. [22], which positions physical literacy holistically as a determinant of health. We utilised the model proposed by Cairney et al. [22], as the basis for a CCA and narrative review with the aims of understanding the influence of the model on, and establishing current trends in, physical literacy and health-focused research; and identifying remaining gaps within the model that warrant further exploration.

The CCA within this narrative review has been a valuable tool for evaluating the efficacy of the Cairney et al. [22] modelled hypothesis that physical literacy is a determinant of physical activity and health. It systematically examined the extent to which key concepts from Cairney et al.’s work has been adopted, applied, or validated in subsequent research. This provides a clear indication of the model’s influence on the academic discourse around physical literacy. By mapping the frequency and context in which Cairney’s model is cited, researchers can assess its impact across different studies, gauging whether it has shaped theoretical frameworks, empirical studies, or interventions related to physical activity and health outcomes.

Moreover, integrating CCA into a narrative review adds depth to the traditional review approach. While narrative reviews synthesise findings across studies to tell a cohesive story, the inclusion of citation analysis enhances this by showing the evolution of scholarly thinking over time. For Cairney et al.’s hypothesis, this is particularly relevant because physical literacy remains an emergent concept, and the field is still solidifying its theoretical foundations. This CCA goes some way to pinpoint shifts in the academic consensus and highlight gaps where further research is needed to solidify the causal links between physical literacy, physical activity, and health.

Cairney et al. [22] was cited in 293 publications, with 16 of these using the included model to inform a theoretical framework; Cairney et al. [22] was predominantly cited in the introduction section of citing publications and cited 2–4 times. That Cairney et al. [22] was frequently cited multiple times indicates a closer relationship to the citing publication [45]. Multiple citations in the methods (n = 5) and results (n = 7) sections indicate that Cairney et al. [22] had a strong influence over the citing publications (more so than an article that only has a single citation in a single section of a publication, for example, the introduction). Given that this paper conducted a more in-depth analysis of only citations that used Cairney et al. [22] to construct a theoretical framework, this result is to be somewhat expected. Only 16 of the 293 citing publications (5.4%) utilised the model proposed by Cairney et al. [22] to inform a theoretical framework. Furthermore, all included articles maintained a neutral disposition of Cairney et al. [22]. It can therefore be concluded that, despite proposing a testable model, open to challenge Cairney et al. [22] is not being used to its full potential within the citing literature. This conclusion is emphasised by the results of the narrative analysis, whereby elements of the model are tested, but the model is not tested as a whole, and population groups on which the model is being tested are limited. Details are provided in the paragraphs that follow.

The results of the narrative analysis demonstrate that physical literacy (both holistically and the individual components) is associated with health-related fitness variables including aerobic fitness (partly mediated by MVPA), body composition, flexibility, and muscular strength and power. Physical literacy was also shown to be associated with total PA and MVPA (± partial mediation by LPA and SB). Furthermore, total physical literacy, movement competence and motivation/confidence were associated with health literacy, and wellbeing. Based on (a) the demonstrated relationships between physical literacy, health-related fitness variables, PA, and health; and (b) the pre-established relationships between health-related fitness and higher levels of PA, reduced risks of cardiometabolic illness/disease and all-cause mortality, and improved mental health and quality of life, [46–51] support exists positioning physical literacy as a health determinant as proposed by Cairney et al. [22].

More than 80% of the available empirical evidence was observational, of which 23% was longitudinal. Given the prominence of cross-sectional designs, the direction of the relationship(s) between physical literacy, PA, and health-related variables remains less clear. If we are to establish how physical literacy can contribute to changes in behavioural or physical, social, and mental health outcomes, more longitudinal studies are required. There are additional critical components of the model that deserve research attention. Examples include testing the mediating effects of PA on the relationship between physical literacy and health; and testing the moderating effects of the physical, social, and psychological adaptations to PA on the relationship between physical literacy and health. Interventional designs that test specific research questions spanning the scope of the model also warrant consideration. For example, whether improved physical literacy results in increased PA, and the resulting impacts on physical, mental, or social health variables.

Gaps also remain in the populations of study. All citing research was conducted on children and young people (< 20 years); however, physical literacy was conceptualized to span across the life course [1, 8–10]. Older adults, for example, represent an interesting population that could benefit from greater physical literacy development as it could be important for maintaining strong mobility and falls prevention. Additionally, the citing literature was predominantly conducted with healthy population groups, despite physical literacy being designed and promoted as an inclusive concept [52]. Research focusing on specific groups (e.g., people living with mental health conditions, cardiometabolic conditions, disabilities etc.) is therefore recommended. Research spanning these population groups will provide increased support for the inclusivity of physical literacy itself, along with critical contributions to understanding the proposed relationship between physical literacy and health outcomes [11].

The varied operationalisation of physical literacy from a measurement perspective was evident across the 16 included citing articles. Sixteen different measures were used to measure total or components of physical literacy, including objective assessor completed measures, and self-report questionnaires. Some studies attempted to assess physical literacy as a holistic concept either perceived (assessed by questionnaire only) or using a combination of assessor completed items along with questionnaires, whereas others measured only certain components of physical literacy (e.g., motor competence). The varied assessment tools utilised reflect a pragmatic, evidence-based approach to measurement [14, 15]. However, measurement in this way (along with the associated fragmented statistical analysis) does not allow for an appreciation of the proposition that physical literacy may be more than a sum of its parts. If we are to understand the relationship between physical literacy and health, it is critical that the physical literacy is measured, and the relationship evaluated pragmatically, yet holistically [53].

## Conclusion

We are currently at a critical juncture for physical literacy-related research. Cairney et al. [22] have attempted to holistically align physical literacy, PA, and health-related outcomes, which has received extensive research attention. Cairney et al. [22] has been cited in 293 publications, though only 16 of these used the model to inform a theoretical framework and therefore ‘tested’ the pathways proposed. There exists some empirical evidence to support certain pathways proposed by Cairney et al. [22], positioning of physical literacy as a determinant of health. This may have significant implications for the prevention and management of chronic health conditions for all individuals. Future research should be focused pragmatically on understanding the mediating and moderating pathways proposed by Cairney et al. [22] to strengthen our current understanding of the relationships between physical literacy (as a holistic construct), PA, and health.

## Data Availability

Data sharing is not applicable to this article as no datasets were generated or analysed during the current study.

## Abbreviations

CCA: Citation content analysis
DCD: Developmental coordination disorder
FMS: Fundamental movement skills
FoR: Field of research codes
JIA: Juvenile idiopathic arthritis
LPA: Low intensity physical activity
MVPA: Moderate-to-vigorous physical activity
PA: Physical activity
SB: Sedentary behaviour
Se: Semantic
Sy: Syntactic
